# Editorial: Genomic discoveries and pharmaceutical development in urologic tumors - volume II

**DOI:** 10.3389/fphar.2025.1743378

**Published:** 2026-01-12

**Authors:** Wenjia Li, Jialin Meng, Huan Yang, Tao Zhang, Lei Yin

**Affiliations:** 1 Department of Cardiovascular Medicine, Ruijin Hospital, Shanghai Jiao Tong University School of Medicine, Shanghai, China; 2 Department of Urology, The First Affiliated Hospital of Anhui Medical University, Anhui Province Key Laboratory of Genitourinary Diseases, Anhui Medical University, Hefei, China; 3 Department of Urology, Tongji Hospital, Tongji Medical College of Huazhong University of Science and Technology, Wuhan, China; 4 Department of Urology, Putuo People’s Hospital, School of Medicine, Tongji University, Shanghai, China; 5 Department of Urology, Putuo People’s Hospital, Tongji University, Shanghai, China; 6 Department of Urology, Shanghai Ninth People’s Hospital, Shanghai Jiao Tong University School of Medicine, Shanghai, China

**Keywords:** urologic tumors, cancer biomarkers, drug screen, drug repurposing, genomic sequence

## Introduction

Urologic malignancies remain a global health concern, with steadily rising incidence and mortality. According to recent epidemiological surveys, prostate cancer has become one of the most common cancers among men worldwide, while bladder and renal cancers continue to pose substantial diagnostic and therapeutic challenges (Albigès et al., 2024; Cicchetti et al., 2025; Kratzer et al., 2025; Leung et al., 2025). The genomic era has profoundly reshaped our understanding of these diseases, unveiling molecular drivers, therapeutic targets, and pathways of drug resistance that now guide personalized treatment strategies.

Building on the success of the first volume of “*Genomic Discoveries and Pharmaceutical Development in Urologic Tumors*”, this second volume broadens the landscape from genetic discoveries to translational and pharmacologic development (Meng et al., 2024). Across ten contributions, investigators integrated genomics, pharmacology, and computational biology to explore novel mechanisms, prognostic signatures, and therapeutic opportunities in prostate, bladder, and renal cancers. Together, these studies form a coherent narrative that bridges mechanistic discovery and clinical translation—the central vision of this Research Topic.

## Genomic and molecular insights in prostate cancer

Five studies in this volume focus on prostate cancer, reflecting its complex genomic and metabolic heterogeneity. Hua et al. investigated SUCLG2, a mitochondrial enzyme associated with the tricarboxylic acid cycle, and found its dysregulation to predict poor prognosis and altered immune infiltration in prostate cancer. Their integration of single-cell RNA sequencing and metabolic pathway analysis highlighted the interplay between tumor metabolism and immune microenvironment, providing a promising metabolic biomarker for prognosis and therapeutic targeting. Wu et al. examined the emerging concept of cuproptosis, a copper-dependent cell-death mechanism, and its relationship with RNA-methylation regulators. By combining single-cell and bulk RNA-seq data, they identified distinct cuproptosis-related subtypes that correlate with immune infiltration and therapeutic response. This multi-omics strategy offers new insights into redox regulation and treatment stratification. Chitluri et al. revealed that inhibition of DPP4 downregulates FGF17 and PDGFRA, suppressing the PI3K/Akt signaling cascade and inducing apoptosis in prostate cancer cells. This work underscores the value of drug repurposing by connecting a clinically used enzyme inhibitor to a novel anti-tumor mechanism. Zhou et al. conducted a comprehensive analysis of lactylation-related genes, integrating single-cell RNA-seq, bulk transcriptomics, and machine learning. They delineated intratumoral heterogeneity in prostate cancer and proposed a lactylation-based prognostic signature that links metabolic reprogramming to tumor aggressiveness and immune escape. Finally, Wang et al. performed a pharmacoepidemiologic study using the WHO-VigiAccess database to characterize adverse-reaction profiles of three anti-prostate-cancer drugs. By combining real-world pharmacovigilance data and bibliometric trends, they provided valuable insights into safety surveillance and rational drug use.

## Molecular mechanisms and therapeutic targeting in bladder cancer

Two articles address the molecular mechanisms and pharmacologic vulnerabilities of bladder cancer. Zhu et al. reported that miR-146b promotes bladder-cancer cell proliferation by targeting SMAD4 and activating the c-Myc/Cyclin D1 axis. This mechanistic dissection not only elucidates tumor-promoting pathways but also provides a rationale for RNA-based therapeutic strategies in high-grade urothelial carcinoma. Complementing this, Yuan et al. combined *in silico* screening, molecular docking, and cell-based assays to identify artesunate—a clinically available anti-malarial—as a multi-target agent that suppresses bladder-cancer growth through MAPK and PI3K/Akt modulation. This integration of computational pharmacology and experimental validation illustrates a productive route for drug repurposing guided by genomic and systems biology.

## Renal tumors and clinical translational advances

Three studies focus on renal cell carcinoma (RCC), where targeted and immune therapies have dramatically transformed treatment paradigms. Li et al. provided a comparative analysis of adverse-event profiles for four multi-targeted tyrosine kinase inhibitors (TKIs) using global pharmacovigilance data. Their study not only identified distinct toxicity spectra for agents such as sunitinib and axitinib but also emphasized the importance of continuous real-world safety monitoring during the post-approval phase. Expanding on the clinical translation dimension, Cui et al. reported the real-world efficacy and safety of tislelizumab plus axitinib as first-line therapy for intermediate- and high-risk metastatic clear-cell RCC. Their data supported the synergistic potential of PD-1 blockade combined with anti-angiogenic therapy, reinforcing the paradigm that rational combination therapy informed by genomic and immune profiling can improve patient outcomes. In addition, Zheng et al. described a rare renal epithelioid neoplasm harboring EWSR1::CREB fusions. This case enriches the spectrum of EWSR1-associated tumors and reminds clinicians of the diagnostic value of next-generation sequencing for rare and morphologically ambiguous renal neoplasms.

In conclusion, *Genomic Discoveries and Pharmaceutical Development in Urologic Tumors–Volume II* continues the mission initiated in Volume I: to connect fundamental genomic discoveries with tangible pharmacologic and clinical applications. From metabolism-linked biomarkers in prostate cancer to repurposed therapeutics in bladder and renal cancers, the ten contributions herein exemplify the multidimensional progress defining modern uro-oncology.

Despite these advances, several key challenges remain. First, a deeper understanding of tumor evolution, clonal dynamics, and therapeutic resistance is required—ideally supported by single-cell and spatial multi-omics technologies. Second, translation from genomic discovery to clinical practice faces persistent obstacles, including patient selection, pharmacokinetic/pharmacodynamic variability, and real-world treatment effectiveness (Yin et al., 2020; Yin et al., 2023; Yoshihara et al., 2025). Third, the integration of immuno-oncology, metabolic reprogramming, and tumor microenvironment analysis into the genomic-driven drug-development pipeline remains an open frontier. Finally, artificial intelligence and big-data analytics will play an increasingly critical role in predicting drug responses, stratifying patients, and optimizing therapeutic combinations (Zou and Green, 2023). Looking forward, international collaboration across genomics, pharmacology, and clinical oncology will be indispensable for realizing the full promise of precision medicine in urologic tumors.
